# Multimorbidity Patterns in a National Representative Sample of the Spanish Adult Population

**DOI:** 10.1371/journal.pone.0084794

**Published:** 2014-01-20

**Authors:** Noe Garin, Beatriz Olaya, Jaime Perales, Maria Victoria Moneta, Marta Miret, Jose Luis Ayuso-Mateos, Josep Maria Haro

**Affiliations:** 1 Research Unit, Parc Sanitari Sant Joan de Déu, Universitat de Barcelona, Sant Boi de Llobregat, Spain; 2 Fundació Sant Joan de Déu, Esplugues de Llobregat, Spain; 3 Instituto de Salud Carlos III, Centro de Investigación Biomédica en Red de Salud Mental, CIBERSAM, Madrid, Spain; 4 Department of Psychiatry, Universidad Autónoma de Madrid, Madrid, Spain; 5 Instituto de Investigación Sanitaria Princesa (IP), Madrid, Spain; INRCA, Italy

## Abstract

**Background:**

In the context of population aging, multimorbidity has emerged as a growing concern in public health. However, little is known about multimorbidity patterns and other issues surrounding chronic diseases. The aim of our study was to examine multimorbidity patterns, the relationship between physical and mental conditions and the distribution of multimorbidity in the Spanish adult population.

**Methods:**

Data from this cross-sectional study was collected from the COURAGE study. A total of 4,583 participants from Spain were included, 3,625 aged over 50. An exploratory factor analysis was conducted to detect multimorbidity patterns in the population over 50 years of age. Crude and adjusted binary logistic regressions were performed to identify individual associations between physical and mental conditions.

**Results:**

Three multimorbidity patterns rose: ‘cardio-respiratory’ (angina, asthma, chronic lung disease), ‘mental-arthritis’ (arthritis, depression, anxiety) and the ‘aggregated pattern’ (angina, hypertension, stroke, diabetes, cataracts, edentulism, arthritis). After adjusting for covariates, asthma, chronic lung disease, arthritis and the number of physical conditions were associated with depression. Angina and the number of physical conditions were associated with a higher risk of anxiety. With regard to multimorbidity distribution, women over 65 years suffered from the highest rate of multimorbidity (67.3%).

**Conclusion:**

Multimorbidity prevalence occurs in a high percentage of the Spanish population, especially in the elderly. There are specific multimorbidity patterns and individual associations between physical and mental conditions, which bring new insights into the complexity of chronic patients. There is need to implement patient-centered care which involves these interactions rather than merely paying attention to individual diseases.

## Introduction

A two-fold increase in the worldwide population over 60 years old is expected between 2006 and 2050 [1]. Aging is associated with an exponential increase in multimorbidity. Two out of three people who have reached retirement age suffer from at least two chronic conditions [2], [3]. Poor clinical and financial outcomes have been observed in individuals with multimorbidity [4]. The negative impact of multimorbidity on clinical outcomes results in poor functional status and high mortality rates, and accounts for 36 million deaths attributed to chronic, non-communicable diseases globally per year [5]–[8]. Associated costs due to chronic conditions reach 75% of total health expenditure, as it is related to the use of a wide variety of health services, such as physician consultation, hospitalization, medication, rehabilitation, long-term care or transportation [9], [10].

When analyzing multimorbidity, most studies have focused on the link between co-occurring pairs of conditions or the mere descriptive counting of diseases [11], [12]. There is a clear need to analyze cumulative interactions between diseases, although few studies have done so [13], [14]. The study of multimorbidity patterns can lead to a deep understanding of multimorbidity. However, in some studies, the individual relationships between diseases were not taken into account but rather the interaction between predetermined domains of diseases, such as the vascular domain or the psychological domain [15]. Associations between these prefixed domains could affect the interpretation of the results since the individual associations between diseases are not assessed. Current population aging trends have led to increasing interest in multimorbidity, resulting in some complex studies on the topic being published [2], [13], [16], [17]. However, they mostly use small sample sizes, are restricted to very elderly participants, cover geographically limited areas or only included patients in primary care settings [2], [13], [18], [19]. In this context, a recent systematic review concluded that there is need for a better description and understanding of multimorbidity [20]. In-depth analysis of multimorbidity may benefit from large-scale population samples, standardized definitions of the diseases considered and statistical methods capable of distinguishing statistically significant associations from spurious ones [18].

Multimorbidity includes both physical and mental conditions. Few studies have analyzed the link between them, or the joint effect of mental and physical conditions on the probability of severe disability [21]–[24]. The reasons for co-occurrence of physical and mental conditions are poorly understood. They may be related to functional disability, pathophysiological mechanisms or cognitive aspects associated with being ill [25]. In the elderly, the study of co-occurrence trends between physical and mental diseases is especially relevant as it is the population with the highest rates of multimorbidity. However, few efforts have been made to study these trends [2], [26].

Recent policy efforts focus on prevention and control of chronic diseases, highlighting the importance of a better understanding of multimorbidity [27]–[29]. At a clinical level, current guidelines mostly focus on individual diseases, ignoring the co-occurrence of other conditions. The need for a broader approach has been stressed, including multimorbidity research, to develop clinical guidelines [30]. In this study, we aim to use a large general population survey to examine:

The distribution of multimorbidity in the adult population.
The multimorbidity patterns in the population over 50 years of age.
The impact of individual physical conditions and multimorbidity on the prevalence of mental conditions in the population over 50 years of age.

## Methods

### Design

This article is based on data from the COURAGE Project, a cross-sectional study of the general non-institutionalized adult population reached through household interviews [31]. The original study included data on populations in three countries: Finland, Poland and Spain. The current analyses are based on data from the Spanish sample.

### Sample and procedures

A stratified, multistage, clustered area probability method was used to select a representative sample of the adult population in Spain. The target group was a community-residing population over 18 years old. Distinct procedures were used to select three samples according to age: 18–49; 50–79; ≥80 years. The populations over 50 years and over 80 years old were oversampled as they were the principal target of the study. People with language barriers were not included in the study. Face-to-face structured interviews were conducted through Computer-Assisted Personal Interviewing (CAPI) at respondents’ homes in 2011 and 2012. The survey protocol was originally designed in English and then translated into Spanish according to WHO translation guidelines for assessment instruments [32]. Lay interviewers were trained with the instruments prior to the administration of the survey. Quality assurance procedures were implemented during fieldwork [33]. The final response rate was 69.9%. At the beginning of the interview, the interviewer judged, subjectively, whether the respondent had cognitive difficulties that would prevent them from answering the questions. In the case of the presence of cognitive problems, a short version of the survey was administered to proxy respondents. Data from proxy respondents were not analyzed since they did not include the diagnoses of all physical and mental conditions. Thus, the final analysis consisted of 4,583 participants, once data from the 170 proxy respondents were eliminated.

### Data collection

Sociodemographic data were obtained with regard to age, gender, education level, marital status, employment status, household income and urbanicity. Chronic physical conditions were assessed by asking the person whether they had received medical diagnosis and treatment during the previous 12 months for angina, arthritis, asthma, cataracts, chronic lung disease, diabetes, edentulism, hypertension or stroke. In addition, questions about specific symptoms were included to detect undiagnosed cases. Algorithms based on clinical symptoms were implemented based on the WHO’s SAGE study, current clinical guidelines and reference publications [34]–[40]. The participant was considered to have a condition if they met at least one of the two previously established criteria for angina, asthma, arthritis, chronic lung disease, stroke or cataracts. Hypertension, diabetes and edentulism had no symptomatic algorithms since they are considered asymptomatic conditions. Previous 12-month mental morbidity (depression and anxiety) was assessed with an adapted version of the World Health Organization Composite International Diagnostic Interview (CIDI), according to DSM-IV criteria.

### Statistical analysis

Unweighted frequencies, weighted proportions, means, confidence intervals and cross tabulations were used for descriptive analysis. The Chi-square test was applied to measure differences in the prevalence of chronic diseases, multimorbidity, number of diseases and sociodemographic variables across age or gender variables.

Multimorbidity patterns were analyzed using exploratory factor analysis in participants over 50 years old. Exploratory factor analysis is a statistical technique used to summarize the correlation among a series of variables, with the expected aim of understanding the underlying structure of the data. This method defines a set of underlying factors, in our case multimorbidity patterns, by estimating the relationship between the variables in each factor. Moreover, it allows distinct variables to be included in various factors. Firstly, a correlation matrix is needed to assess the correlation structure between the variables; chronic conditions in our study. The tetrachoric correlation matrix was used due to the dichotomous nature of the variables, so that it is assumed that diseases included in our analysis have a progressive course and are diagnosed when they reach a certain threshold [41]. By using the results of the tetrachoric correlation matrix, the factor analysis technique leads to a certain number of factors but a selection of the statistically relevant ones is needed. The number of factors extracted corresponded to those with an eigenvalue of at least 1.0 [2]. For every selected factor, there is a factor loading value corresponding to each of the variables. A specific condition was selected to form part of a pattern if its corresponding factor loading was above 0.25, which indicates a stronger association [2], [18]. The Kaiser-Meyer-Olkin method was used to estimate the adequacy of the sample in the factor analysis, whilst cumulative variance was determined to describe the variance of the diagnostic data explained by the pattern. An oblique rotation (Oblimin) was performed to allow a better interpretation of the analysis factor.

Crude and adjusted binary logistic regressions were used to examine the relationship between physical conditions/physical multimorbidity with depression and anxiety in participants over 50 years old. Adjusted models included age, gender, education level, marital status, urbanicity and number of physical conditions. Results are reported as unadjusted and adjusted odds ratios (OR) with 95% CI.

Weights were used in all analyses to adjust for differential probabilities of selection within households, and post-stratification weights to match the samples to population socio-demographic distributions. The statistical analyses took into account the complex sampling design except for multimorbidity patterns, as this analysis was not available in the statistical packages for complex samples. Analyses were performed using IBM SPSS statistics 19 and STATA version 12.

### Ethics statement

The COURAGE study was approved by the partners’ Ethics Committees: Ethics Review Committee Fundació Sant Joan de Déu, Barcelona, Spain and Ethics Review Committee, La Princesa University Hospital, Madrid, Spain. Written informed consent was obtained from the participants and all investigators proceeded according to the principles expressed in the Declaration of Helsinki.

## Results

### Participant characteristics

The study population consisted of 4,583 participants. Statistically significant differences were detected when comparing age groups (18–49; 50–64; ≥65 years) with regard to education level, gender, household income, marital status and employment status, but not for urbanicity (Table 1). Lower educational levels and household incomes were linked to older participants. A higher proportion of women was observed in participants over 65 years.

**Table 1:** Description of the sample of the Spanish Cohort of the COURAGE study.

		Total sample (n = 4583)	18–49 years	50–64 years	≥65 years	p
**Age (mean; se)**		47.6 (0.3)	35.7 (0.3)	57.0 (0.1)	74.9 (0.1)	
**Education (n; %)**						<0.001
	No education	1269 (16.6%)	62 (6.5%)	311 (16.6%)	896 (46.7%)	
	Primary	1265 (24.9%)	190 (20.9%)	548 (32.8%)	527 (29.8%)	
	Secondary	1423 (39.3%)	474 (48.1%)	638 (35.4%)	311 (16.6%)	
	University+	625 (19.2%)	232 (24.5%)	263 (15.2%)	130 (6.8%)	
**Gender (n; %)**						0.006
	Male	2078 (49.4%)	435 (51.4%)	829 (47.7%)	814 (45.0%)	
	Female	2505 (50.6%)	523 (48.7%)	931 (52.3)	1051 (55.0%)	
**Household income (n; %)**						<0.001
	1st quintile	871 (22.5%)	185 (22.8%)	357 (23.2%)	329 (21.0%)	
	2nd quintile	804 (16.3%)	110 (12.9%)	268 (17.9%)	426 (25.0%)	
	3rd quintile	875 (20.0%)	160 (19.0%)	273 (17.1%)	442 (25.6%)	
	4th quintile	962 (22.8%)	217( 23.5%)	370 (22.4%)	375 (21.0%)	
	5th quintile	624 (18.4%)	196 (21.8%)	303 (19.4%)	125 (7.4%)	
**Marital status (n; %)**						<0.001
	Single	667 (27.3%)	357 (39.2%)	187 (10.4%)	123 (6.8%)	
	Married	2777 (56.6%)	519 (53.1%)	1238 (71.1%)	1020 (54.2%)	
	Separated/divorced	342 (7.1%)	76 (7.2%)	196 (10.9%)	70 (3.4%)	
	widowed	797 (9.0%)	6 (0.5%)	139 (7.6%)	652 (35.7%)	
**Urban pattern (n; %)**						0.216
	Urban	3958 (84.8%)	820 (85.4%)	1523 (83.7%)	1615 (83.9%)	
	Rural	625 (15.2%)	138 (14.6%)	237 (16.3%)	250 (16.1%)	
**Employment (n; %)**						<0.001
	Working	1360 (46.2%)	543 (61.3%)	773 (47.5%)	44 (2.3%)	
	Retired	1387 (16.4%)	2 (0.2%)	183 (11.4%)	1202 (66.6%)	
	Other	1611 (37.4%)	342 (38.4%)	681 (41.2%)	588 (31.2%)	

SE =  Standard Error.

Note =  unweighted frequencies (n), and weighted means and proportions are displayed. Household income was divided into 5 quintiles. Education category ‘no education’ included those people that had never been to school or did not finish primary school. Marital status ‘married’ category included currently married or cohabiting. Employment ‘other’ category included training, homemakers, unemployed, voluntary work, health problems, caring for family, sick leave, no need to work, temporary time off and voluntary work.

### Chronic conditions and multimorbidity prevalence in the overall population

In the overall population, hypertension, arthritis and cataracts were the most common conditions with prevalences of 16.6%, 13.7% and 10.6%, respectively (Table 2). Depression was the most prevalent of the mental disorders assessed, with a prevalence of 9.0%. Multimorbidity occurred in 20.0% of the sample, whilst 4.8% of the participants suffered from four or more conditions (Table 3). Differences in the prevalences and multimorbidity were detected according to age and gender. Prevalence of individual conditions, multimorbidity and number of chronic conditions were higher in the population over 65 except for anxiety, which was not statistically significant. Women had higher rates of depression, cataracts, arthritis, anxiety, multimorbidity and overall number of chronic conditions. Women over 65 years old represent the population subgroup with the highest multimorbidity rate (67.3%). In this subgroup, hypertension, arthritis, cataracts and depression accounted for 53.8%, 45.7%, 43.1% and 18.0% of the participants, respectively.

**Table 2:** Prevalence of 12-month physical conditions, mental disorders and multimorbidity according to age and gender.

	TOTAL	18–49 years		50–64 years		≥ 65 years		p (age)	p (sex)
		Men	Women	Men	Women	Men	Women		
	% (95% CI)	% (95% CI)	% (95% CI)	% (95% CI)	% (95% CI)	% (95% CI)	% (95% CI)		
**Depression**	9.0 (7.9–10.2)	5.2 (3.5–7.5)	9.0 (6.8–11.7)	8.4 (6.4–11.1)	15.2 (11.6–19.7)	5.1 (3.7–6.8)	18.0 (15.6–20.6)	<0.001	<0.001
**Angina**	2.8 (2.4–3.3)	0.4 (0.2–0.9)	0.5 (0.1–1.6)	5.3 (3.7–7.6)	2.4 (1.6–3.7)	10.1 (8.4–12.0)	8.5 (6.5–10.9)	<0.001	0.337
**Cataracts**	10.6 (9.9–11.4)	1.7 (0.9–3.3)	2.0 (1.1–3.5)	7.8 (6.4–9.6)	9.7 (8.0–11.7)	32.3 (29.2–35.6)	43.1 (39.9–46.5)	<0.001	<0.001
**Asthma**	4.8 (4.1–5.7)	3.6 (2.3–5.7)	4.2 (2.9–5.9)	3.6 (2.6–4.9)	5.0 (3.7–6.9)	7.1 (5.4–9.3)	8.8 (6.9–11.2)	<0.001	0.178
**Hypertension**	16.6 (15.5–17.7)	4.1 (2.4–6.7)	2.7 (1.5–4.7)	25.8 (22.9–29.0)	21.3 (18.2–24.7)	44.8 (40.7–48.9)	53.8 (50.6–56.9)	<0.001	0.066
**Edentulism**	9.0 (7.7–10.6)	3.2 (1.7–5.9)	2.5 (1.2–4.9)	9.2 (7.4–11.3)	7.3 (5.5–9.6)	24.9 (21.5–28.7)	31.0 (26.6–35.7)	<0.001	0.081
**Diabetes**	6.7 (6.0–7.5)	2.5 (1.5–4.2)	1.5 (0.7–3.1)	11.4 (9.3–14.0)	7.7 (5.8–10.1)	18.6 (16.1–21.5)	17.6 (15.4–20.1)	<0.001	0.219
**Arthritis**	13.7 (12.7–14.8)	3.2 (1.8–5.6)	7.7 (5.9–10.0)	10.9 (8.8–13.4)	25.3 (22.1–28.7)	20.4 (17.6–23.6)	45.7 (41.9–49.5)	<0.001	<0.001
**Chronic Lung D**	3.4 (2.8–4.0)	1.3 (0.6–2.8)	1.1 (0.6–2.3)	4.8 (3.4–6.7)	3.3 (2.3–4.8)	11.5 (9.1–14.6)	7.2 (5.7–9.1)	<0.001	0.100
**Stroke**	2.1 (1.7–2.6)	0.7 (0.3–1.9)	0.4 (0.1–1.2)	2.4 (1.4–4.0)	2.3 (1.4–3.8)	7.5 (5.2–10.7)	5.9 (4.2–8.3)	<0.001	0.509
**Anxiety**	1.1 (0.8–1.5)	0.8 (0.3–2.5)	1.0 (0.4–2.4)	1.2 (0.7–2.1)	2.4 1.6–3.5)	0.1 (0.1–0.8)	1.8 (1.1–2.8)	0.120	0.154
**Multimorbidity**	20.0 (18.8–21.2)	4.0 (2.7–5.9)	6.0 (4.2–8.4)	21.3 (18.2–24.6)	27.0 (23.2–31.3)	52.9 (48.7–57.0)	67.3 (64.6–70.0)	<0.001	<0.001

CI =  Confident interval. Note =  Weighted proportion and 95% Confident Intervals are shown. Anxiety included Generalised Anxiety Disorder and Panic Disorder. Multimorbility is defined as the presence of ≥ 2 physical or mental conditions. p(age) refers to statistical differences in the three groups of age; p(sex) refers to differences in gender, regardless the age group.

**Table 3:** Number of total, physical and mental conditions according to age and gender.

		TOTAL	18–49 years		50–64 years		> 65 years		p (age)	p (sex)
			Men	Women	Men	Women	Men	Women		
		% (95%CI)	% (95%CI)	% (95%CI)	% (95%CI)	% (95%CI)	% (95%CI)	% (95%CI)		
**Total conditions**									<0.001	<0.001
	0	58.7 (56.9–60.6)	78.1 (73.9–81.9)	76.4 (72.6–79.8)	47.5 (43.7–51.3)	45.2 (41.2–49.2)	20.7 (17.0–25.0)	9.9 (8.6–11.4)		
	1	21.3 (19.7–23.0)	17.91 (14.5–22.0)	17.7 (14.5–21.4)	31.3 (28.1–34.7)	27.8 (23.5–32.6)	26.4 (23.6–29.4)	22.8 (20.3–25.5)		
	2	9.7 (9.0–10.6)	3.36 (2.2–5.0)	4.37 (3.0–6.3)	11.1 (9.15–13.4)	15.4 (12.6–18.7)	24.4 (21.2–28.0)	23.8 (21.2–26.6)		
	3	5.4 (4.9–6.1)	0.64 (0.2–2.1)	0.90 (0.3–2.7)	5.5 (4.1–7.4)	6.4 (4.8–8.6)	15.9 (132–19.1)	21.1 (18.6–24.0)		
	≥4	4.8 (4.3–5.4)	0	0.69 (0.2–2.1)	4.7 (3.1–7.0)	5.2 (4.13–6.60)	12.6 (10.4–15.1)	22.4 (19.7–25.4)		
**Physical conditions**									<0.001	<0.001
	0	62.5 (60.7–64.2)	82.5 (78.5–85.8)	81.3 (78.1–84.1)	49.5 (45.7–53.3)	51.6 (46.5–56.5)	20.8 (17.1–25.1)	10.8 (9.5–12.3)		
	1	20.1 (18.6–21.7)	14.9 (11.7–18.8)	16.4 (13.7–19.6)	31.6 (28.5–34.9)	26.6 (23.4–30.0)	27.4 (24.7–30.4)	24.2 (21.9–26.8)		
	2	8.5 (7.8–9.3)	2.2 (1.4–3.7)	1.36 (0.76–2.4)	10.8 (8.8–13.3)	12.9 (10.6–15.8)	24.1 (20.9–27.6)	26.4 (23.7–29.3)		
	≥3	8.9 (8.3–9.6)	0.4 (0.1–1.7)	0.91 (0.4–2.4)	8.05 (6.1–10.6)	8.9 (7.3–10.9)	27.8 (24.1–31.7)	38.6 (35.6–41.6)		
**Mental conditions**									<0.001	<0.001
	0	90.5 (89.3–91.7)	94.1 (91.6–95.8)	90.7 (87.8–93.0)	91.3 (88.6–93.4)	84.3 (79.7–88.0)	94.8 (93.0–96.2)	81.6 (78.9–84.0)		
	≥1	9.5 (8.4–10.7)	5.9 (4.2–8.4)	9.3 (7.0–12.2)	8.7 (6.6–11.4)	15.7 (12.0–20.3)	5.2 (3.8–7.0)	18.4 (16.0–21.1)		

CI =  Confident interval. Note =  Weighted proportion and 95% Confident Intervals are shown.

### Multimorbidity patterns in older adults

In the factor analysis, three factors were selected according to the results of the eigenvalues. The adequacy of the sample was considered acceptable with a KMO value of 0.70, and a cumulative variance of 39.5%. The first multimorbidity pattern included angina, asthma and chronic lung disease (cardio-respiratory factor). The second one included arthritis, anxiety and depression (mental-arthritis factor) (table 4). Finally, the third pattern included hypertension, angina, stroke, diabetes, cataracts, arthritis and edentulism (aggregate pattern). All conditions were related to at least one pattern. Angina and arthritis were both present in two multimorbidity patterns.

**Table 4:** Factor score for each condition in participants over 50 years.

Condition	Factor1	Factor2	Factor3
**Angina**	**0.40**	0.24	**0.33**
**Cataracts**	0.20	0.20	**0.48**
**Asthma**	**0.80**	0.10	0.05
**Hypertension**	0.15	0.10	**0.50**
**Edentulism**	0.09	0.10	**0.37**
**Diabetes**	0.17	0.01	**0.50**
**Arthritis**	0.24	**0.27**	**0.28**
**Chronic Lung Disease**	**0.79**	0.15	0.07
**Stroke**	0.09	0.04	**0.46**
**Anxiety**	0.07	**0.78**	–0.01
**Depression**	0.24	**0.76**	0.12

Factor 1 (cardio-respiratory); Factor 2 (mental-arthritis); Factor 3 (aggregate pattern).

Note =  Factor scores ≥0.25 are highlighted.

### Association between physical and mental conditions in older adults

Table 5 shows the crude and adjusted logistic regression between physical and mental conditions in a sample of people over 50. For crude analysis, all physical conditions but stroke were associated with depression. Arthritis, angina, chronic lung disease and asthma still remained associated with depression after adjusting for covariates. With regard to the number of physical conditions, patients with two and those with three or more physical conditions were at higher risk of suffering from depression compared with participants without any physical conditions (adjusted OR: 2.24, CI: 1.33–3.74; adjusted OR 4.38, CI: 2.31–8.33). Crude logistic regression linked angina, edentulism and arthritis with anxiety. After adjusting for covariables, the association with angina remained statistically significant. Having three or more physical conditions was also related to suffering from anxiety (adjusted OR 5.23, CI: 1.76–15.53).

**Table 5:** Logistic regression models to predict mental conditions.

	Depression model		Anxiety model	
	OR crude (95%CI)	AOR (95%CI)	OR crude (95%CI)	AOR (95%CI)
**Asthma**	**2.79 (2.01**–**3.85)**	**1.86 (1.31**–**2.64)**	1.60 (0.67–3.84)	0.90 (0.37–2.19)
**Cataracts**	**1.99 (1.47**–**2.68)**	1.32 (0.95–1.83)	1.64 (1.00–2.71)	1.17 (0.66–2.08)
**Angina**	**2.55 (1.80**–**3.61)**	**2.01 (1.40**–**2.90)**	**3.96 (2.25**–**6.98)**	**3.39 (1.84**–**6.22)**
**Edentulism**	**1.43 (1.09**–**1.88)**	0.96 (0.74–1.24)	**1.73 (1.03**–**2.91)**	1.38 (0.80–2.37)
**Hypertension**	**1.50 (1.10**–**2.03)**	0.92 (0.70–1.21)	1.51 (0.85–2.68)	0.94 (0.50–1.77)
**Chronic Lung Disease**	**3.18 (2.21**–**4.59)**	**2.66 (1.84**–**3.86)**	2.09 (0.87–5.10)	1.49 (0.56–3.97)
**Stroke**	1.77 (0.97–3.24)	1.30 (0.72–2.34)	0.84 (0.34–2.10)	0.59 (0.22–1.57)
**Arthritis**	**2.70 (1.98**–**3.69)**	**1.62 (1.19**–**2.21)**	**2.64 (1.52**–**4.59)**	1.46 (0.73–2.90)
**Diabetes**	**1.73 (1.23**–**2.43)**	1.25 (0.85–1.84)	0.82 (0.44–1.54)	0.54 (0.28–1.04)
**Number of physical conditions**				
**0**	1	1	1	1
**1**	1.29 (0.68–2.46)	1.41 (0.75–2.67)	1.21 (0.43–3.43)	1.43 (0.48–4.25)
**2**	**1.95 (1.14**–**3.35)**	**2.24 (1.33**–**3.74)**	2.13 (0.76–5.97)	2.94 (1.00–8.67)
**3+**	**3.72 (1.94**–**7.13)**	**4.38 (2.31**–**8.33)**	**3.50 (1.31**–**9.33)**	**5.23 (1.76**–**15.53)**

OR =  Odds Ratio; 95%CI =  Confident interval; AOR = Adjusted Odds Ratio; in bold, statistically significant (p<0.05).

Note = Adjusted models included age, gender, education level, marital status, urban pattern and number of physical conditions.

## Discussion

The results of our study revealed three multimorbidity patterns in the population over 50 years old. The identification and analysis of these patterns is important as very high multimorbidity rates have been detected in this population group. Furthermore, relationships between certain physical and mental conditions have been detected, which is also important for better understanding and management of these diseases.

The first multimorbidity pattern, “cardio-respiratory”, included angina, chronic lung disease and asthma. A recent systematic review found an increased risk of cardiovascular disease in COPD patients [42]. In fact, the presence of obstruction, restriction and respiratory symptoms have been found to be related to higher risk of cardiovascular disease, even after adjusting for other conditions [43]. In addition to having smoking as a common risk factor, the relationship between cardiovascular and chronic pulmonary diseases may involve systemic inflammation, oxidative stress, hypoxia or aging [43], [44]. Atherosclerosis is thought to be closely connected to lipid metabolism but also to inflammation [45]. In COPD there is a pro-inflammatory systemic state which may exacerbate the atherosclerotic process and its consequent negative cardiovascular effects. Moreover, at the diagnostic level there is an overlap in symptoms in this pattern, such as shortness of breath or chest pain, which may be important in the management of these patients.

The second multimorbidity pattern, “mental-arthritis”, includes depression, anxiety and arthritis. Anxiety and depressive disorders are known to be comorbid in many cases. The NESDA study found that 63% of patients with current anxiety disorders had a current depressive disorder and 81% had a lifetime depressive disorder [46]. Additionally, in the ESEMeD study, suffering from general anxiety disorder or panic disorder was clearly associated with a higher risk of major depression [47]. Our results, linking arthritis to psychiatric disorders, support the results found in the World Mental Health Surveys across 17 different countries where arthritis resulted in a higher risk of developing mood disorders and anxiety disorders [48]. Moreover, comorbid depression-anxiety was found to be more strongly associated with arthritis than single mental disorders, which also supports our results [49]. Even though the specific mechanism underlying this relationship still remains unclear, longitudinal data suggest that arthritis would predict the new onset of psychiatric disorders [50].

The third pattern, artificially named the “aggregate pattern”, is a broader one including seven physical conditions. Angina, hypertension, diabetes and stroke are related through the metabolic syndrome. Cataracts may be involved in this pattern as it is influenced by diabetes but also has been linked to joint diseases [2]. The underlying mechanisms that may exist between joint diseases and cataracts are unclear. Adverse effects of glucocorticoid for the treatment of rheumatism could be partially responsible for the higher prevalence of cataracts in these patients. However, Falsarella et al (2013) found a higher risk of developing cataracts after adjusting for glucocorticoid intake in patients with arthritis [51]. Thus, it has been suggested that an increase in inflammatory modulators in rheumatic disorders may also be related to the onset of cataracts [51]. Heart diseases have also been associated with joint diseases, which supports this pattern, and may be linked through inflammatory pathways [2], [18]. A systematic review found a relationship between the presence of edentulism with hypertension, coronary artery disease, diabetes, rheumatoid arthritis and osteoporosis [52]. It has been suggested that edentulism may be related to arthritis through an inflammatory pathway and with cardiovascular diseases through dietary or inflammatory causes [52].

Regarding the relationship between physical and mental conditions, asthma, angina, chronic lung disease and arthritis were associated with depression in the binary logistic regressions after adjusting for covariates. Only angina showed a clear association with anxiety after adjusting for covariates. These associations have been highlighted in previous studies [53]–[56]. There are some hypotheses to explain these findings. Firstly, arthritis, angina, chronic lung disease and asthma present with unpleasant symptoms such as joint pain, chest pain or shortness of breath, whereas cataracts, diabetes, hypertension, edentulism or stroke are mainly asymptomatic [25], [57]. Moreover, these diseases may be linked to higher disability, leading to isolation or frustration [58]. Other explanations include the possible effects of pro-inflammatory cytokines, platelet activation, disturbances in the autonomic nervous system or hypothalamic-pituitary-adrenal axis dysfunction [53]. It should be noted that previous studies also found other relationships, i.e., diabetes with depression [49]. Further research is needed to assess the directionality of these effects and confirm other specific relationships. In addition to co-occurring pairs, the number of physical conditions was also associated with higher prevalence rates of depression and anxiety. The small amount of evidence that exists, is in agreement with our findings [13].

Multimorbidity was present in 20.0% of the overall adult population, consistent with results of about 20.3–30% from similar studies [59]–[61]. An increase in the prevalence of multimorbidity was associated with age, reaching 67.3% in women and 52.9% in men over 65 years, which is also comparable to results described in recent reviews [20], [62]. In fact, most studies on multimorbidity in the literature focus on the old or very-old population, the subgroups with the highest rates. However, in our study, multimorbidity was found in more than 20% of the subgroup between 50–64 years. This result highlights the importance of using a broader age framework to achieve deeper understanding of the phenomenon. Multimorbidity was also related to gender, with women suffering from more overall conditions, physical conditions and mental conditions than men. These results are consistent with most multimorbidity studies [20]. Once examined individually, depression, cataracts and arthritis showed statistically significant differences across gender. These results show that special attention should be paid to the management of elderly women in health care as they are more prone to develop multimorbidity and the effect on the quality of life is more severe than men [63].

Regarding the individual prevalence of chronic physical conditions, hypertension, arthritis and cataracts were the most prevalent physical conditions in older adults, affecting over 40% in the 65+ subgroup. The high prevalence of cardiovascular-related conditions should be highlighted, as they are the second cause of premature mortality in Spain after cancer [64]. Edentulism was present in 24.9% of men and 31.0% of women over 65 years. This value is relevant because edentulism is related to poorer quality of life but also represents an indicator of the adequacy of the national oral health care system [65], [66]. Chronic lung disease and asthma also showed significant increases across age. There is controversy regarding the prevalence of asthma in the elderly. It is assumed that asthma prevalence may decrease with age but some studies suggest underdiagnosis due to diagnostic difficulties [67], [68]. Our results are consistent with the last national health survey in Spain, which showed the highest prevalence of asthma in the population over 85 years [69]. The overlapping symptoms between late onset asthma and chronic obstructive pulmonary disease could be partially present in our results, so that caution is required when interpreting this outcome. According to our results, older adults and elderly people often suffer from chronic diseases which can be partially prevented, e.g., diabetes, angina, chronic lung disease, so that further efforts must be made in our country to develop appropriate national health policies. Once established, tight control of some of these conditions is associated with better health outcomes. Thus, it is essential to maximize their management, which is especially important due to the high prevalence of conditions such as diabetes or hypertension.

Interesting results arise when comparing the prevalence of mental conditions across age. No difference was found in the prevalence of anxiety when comparing age groups. Prevalence of depression showed differences across the three groups. There is controversy surrounding the prevalence of mental disorders in the elderly. The ESEMeD study found a decrease in the prevalence of 12-month anxiety and mood disorders across age [70]. In the ESEMeD study, prevalence of any mental disorder in the last 12 months was lower than in our case, 9.8% in the 50–64 group and 5.8% in the 65+ group. By contrast, some studies have shown much higher prevalence [71], [72]. These differences may be explained in various ways. It has been pointed that the elderly have to cope with several issues which could be related to higher incidence of mental disorders: cognitive decline, sensory impairment, decrease in social relationships, cessation of activity and change of status [73]. Economic recession is also a factor related to the current higher prevalence of mental disorders in Spain [74]. On the other hand, differences in prevalence may be partially explained by different approaches according to the diagnosis scheme of the study. This especially affects the results in the elderly because lower prevalence of depression may be due to the excessive cognitive requirements of the diagnostic interviews, and the attribution of the symptoms to physical illnesses in the elderly [75]. This problem will be addressed with new tools such as a specific version of the CIDI for the population over 65, currently in preparation [76].

Our study has several limitations. The cross-sectional nature of the study may affect the interpretation of the results, so that longitudinal studies are needed to better understand associations. This kind of study does not distinguish between age effects and cohort effects. Moreover, study of multimorbidity would benefit from standardized inclusion and conceptualization of diseases [2], [20]. Studies with a similar number but different conditions assessed make comparison difficult [2]. Some studies have taken a broader approach using the Expanded Diagnosis Clusters (EDC) of the ACG® system, which are more exhaustive but complex to conduct outside the clinical settings or in the case of poor integration between health care levels [18]. A higher number of included conditions logically results in a higher proportion of multimorbidity [20]. Furthermore, when counting diseases, they were scored independently of severity, which can also introduce bias. In our case, the selection of the conditions was done according to the World Health Organization’s SAGE study. SAGE’s inclusion criteria focused on a limited number of conditions impacting significantly on health that is general enough to work with across countries. There is, however, a need to include the diagnosis of dementia in future studies, as it is a common condition in the elderly which has a considerable impact on quality of life, disability and health care resources. The self-reported data collection method could also bias the results. However, this effect may be minor since previous studies have found a good correlation between self-reported and medical-record diagnoses [77], [78]. Finally, our study did not include the medication list or the current number of drugs taken by the patient. Since multimorbidity is intimately related to polypharmacy and inappropriate drugs can have a considerable impact on the health of the elderly, it would be useful to include this information in future studies.

## Conclusions and Future Research

The results of our study contribute to a deeper understanding of chronic conditions and multimorbidity at various levels. In Spain, multimorbidity reaches a considerable prevalence in adults over 65 years old, but also in patients between 50 and 64. Several multimorbidity patterns and relationships between physical and mental conditions have been detected. The knowledge of these associations could lead to an integrated approach to patients suffering from these diseases, both from a clinical and a public health perspective. Patients with multimorbidity are more complex and require a greater number of medical consultations. Integrated plans taking multimorbidity into account represent an opportunity to improve the cost-efficiency of the health care system. Further research with a longitudinal approach is needed to assess the causes, the clinical impact and the financial implications of these associations.
