# Correction: Multimorbidity Patterns in a National Representative Sample of the Spanish Adult Population

**DOI:** 10.1371/journal.pone.0123037

**Published:** 2015-04-02

**Authors:** The PLOS ONE Staff

There is information missing from the Funding section. The correct Funding statement is as follows: The research leading to these results has received funding from the European Community’s Seventh Framework Programme (FP7/2007-2013) under grant agreement number 223071 (COURAGE in Europe) and the Spanish Ministry of Education, Culture and Sport (FPU12/05661). Additional support was provided by funds, cofinanced with FEDER, from the Instituto de Salud Carlos III-FIS research grants number PS09/00295, PS09/01845 and PI12/01490, from the Spanish Ministry of Science and Innovation’s ACI-Promociona (ACI2009-1010). The study was supported by the Centro de Investigación Biomédica en Red de Salud Mental (CIBERSAM), Instituto de Salud Carlos III. This article is part of a PhD programme developed in the Public Health Department of the Universitat de Barcelona, Spain. The funders had no role in study design, data collection and analysis, decision to publish, or preparation of the manuscript.
